# Hyperoxia-activated Nrf2 regulates ferroptosis in intestinal epithelial cells and intervenes in inflammatory reaction through COX-2/PGE2/EP2 pathway

**DOI:** 10.1186/s10020-024-00993-7

**Published:** 2025-01-03

**Authors:** Yanping Liu, Tianming Li, Changping Niu, Zhengwei Yuan, Siyu Sun, Dongyan Liu

**Affiliations:** 1 Department of Gastroenterology and Medical Research Center, Liaoning Key Laboratory of Research and Application of Animal Models for Environmental and Metabolic Diseases, ShengJing Hospital of China Medical University, SanHao Street No. 36, HePing District, Shenyang, 110000 Liaoning China; 2 Department of Gastroenterology, ShengJing Hospital of China Medical University, SanHao Street No. 36, HePing District, Shenyang, 110000 Liaoning China; 3 Laboratory of Health Ministry for Congenital Malformation, ShengJing Hospital of China Medical University, SanHao Street No. 36, HePing District, Shenyang, 110000 Liaoning China; 4 Department of Pathology, Cancer Hospital of China Medical University, Cancer Hospital of Dalian University of Technology, Liaoning Cancer Hospital & Institute, No. 44 Xiaoheyan Road, Dadong District, Shenyang, 110042 Liaoning People’s Republic of China

**Keywords:** Hyperoxia, Intestinal epithelial cells, Ferroptosis, COX-2/PGE2/EP2 pathway, Inflammatory

## Abstract

The lack of knowledge about the mechanism of hyperoxia-induced intestinal injury has attracted considerable attention, due to the potential for this condition to cause neonatal complications. This study aimed to explore the relationship between hyperoxia-induced oxidative damage and ferroptosis in intestinal tissue and investigate the mechanism by which hyperoxia regulates inflammation through ferroptosis. The study systematically evaluated the effects of hyperoxia on oxidative stress, mitochondrial damage, ferroptosis, and inflammation of intestinal epithelial cells both in vitro and in vivo. The results showed that ferroptosis was involved in intestinal oxidative damage caused by hyperoxia and was regulated by Nrf2. Moreover, hyperoxia-induced oxidative damage regulated inflammation through ferroptosis by upregulating the COX-2/PGE2/EP2 signaling pathway. These findings have important implications for future clinical prevention and therapeutic approaches to neonatal organ injury caused by hyperoxia treatment.

## Introduction

Perinatal asphyxia leading to hypoxia is one of the main causes of neonatal morbidity and mortality, often requiring oxygen inhalation therapy (hyperoxia). However, hyperoxia for long time has been proven to be toxic (Ottolenghi et al. 2020), causing damage to neonatal lung, retina, and nerve development (Zhang et al. 2022a; Podraza 2020; Eslami et al. 2021). Researchers have also recently focused on the intestinal injury caused by hyperoxic toxicity. A previous study showed that after neonatal exposure to hyperoxia, the number of goblet cells in the intestines decreased, and the mucus layer was severely damaged, leading to higher intestinal permeability and bacterial translocation (Chou and Chen 2017). Moreover, under hyperoxia, there was a significant increase in the levels of interferon-gamma (IFN-γ) and interleukin 10 (IL-10), indicating intestinal injury (Liu et al. 2020). Given that the development of the intestines plays a crucial role in early-stage nutrient absorption, it is important to investigate the effects and molecular mechanisms of hyperoxic toxicity on the newborn intestine.

Under physiological conditions, the amount of reactive oxygen species (ROS) is balanced with antioxidant defense, precisely controlled by cells to avoid oxidative stress and cell damage (Zeng et al. 2023). However, under hyperoxia, ROS increases, and oxidative stress occurs in cells when ROS exceeds the antioxidant capacity (Fernandes et al. 2021). In a previous study, we found that hyperoxia induced intestinal epithelial cells to release a large amount of ROS, leading to cell death, indicating that excessive ROS caused by hyperoxia is a major cause of oxidative damage (Zhao et al. 2018). Current evidence suggests that ferroptosis is a form of oxidative damage characterized by decreased antioxidant capacity and ROS accumulation (Yang and Yang 2022). Therefore, we speculate that ferroptosis is involved in hyperoxia-induced intestinal oxidative damage.

Ferroptosis is a newly discovered form of cell death caused by the disorder of redox state in intracellular environment controlled by glutathione peroxidase 4(GPX4) (Jiang et al. 2021). When ferroptosis occurs, the antioxidant capacity of cells decreases, and ROS accumulates, resulting in lipid peroxidation of the cell membrane. Currently, most studies on ferroptosis focus on malignant tumors and degenerative diseases, with mechanistic studies primarily focusing on abnormalities in iron metabolism, lipid peroxidation, and the Kelch-like ECH-associated protein 1(Keap1)-Nuclear factor E2-related factor 2 (Nrf2) pathway (Liang et al. 2022; Park et al. 2021; Li et al. 2021). Furthermore, investigations into ferroptosis in intestinal diseases mainly included intestinal ischemia/reperfusion injury and inflammatory bowel disease (Li et al. 2019; Chen et al. 2021). However, there have been no studies on ferroptosis in the process of hyperoxia-induced oxidative damage of intestinal tissue.

Nrf2 is a transcription factor that plays a crucial role in the antioxidant process. It has been reported that Nrf2 can regulate ferroptosis in several ways, such as fighting ferroptosis through its targeting gene-ferritin heavy chain (FTH1) (Yuan et al. 2021). When cells are exposed to a ferroptosis inhibitor, the Keap1-Nrf2 pathway is activated, thereby reducing sensitivity to ferroptosis (Luo et al. 2022). Additionally, Nrf2 can eliminate ROS through glutathione (GSH) metabolism and regulate the ROS level by controlling the steady flow of free ferrous ions (Yang et al. 2022). Our previous research showed that in hyperoxia Nrf2 increased to neutralize much ROS in intestinal epithelial cells (Liu et al. 2021). So Nrf2 might inhibit ferroptosis to protect intestinal epithelial cells in hyperoxia.

Ferroptosis not only promotes cell death but also intensifies the inflammatory reaction by releasing damage-associated molecular patterns (DAMPs) (Sun et al. 2020). This has been supported by the studies demonstrating the anti-inflammatory effect of ferroptosis inhibitors in animal models of many diseases (Shen et al. 2020; Linkermann et al. 2014; Chen et al. 2019). COX-2 is a key inflammatory mediator that regulates inflammation, cell proliferation, and angiogenesis by synthesizing prostaglandins (PGs) and thromboxanes from arachidonic acid (AA) (Dagallier et al. 2021). Prostaglandin E2 (PGE2) is synthesized by all human cells and plays a complex role in inflammation (Ricciotti and FitzGerald 2011). Yang et al. discovered that ferroptosis directly increases the expression of prostaglandin-endoperoxide synthase 2 (PTGS2) encoding cyclooxygenase-2(COX-2), accelerates the metabolism of AA, and promotes the secretion of inflammatory signal molecules (Yang et al. 2014). Moreover, clinical studies have shown that dietary polyunsaturated fatty acids (PUFAs), especially AA, can cause intestinal inflammatory diseases such as Crohn's disease by promoting ferroptosis (Xu et al. 2020; Wang et al. 2020). Hence, it is plausible that hyperoxia induces both ferroptosis and COX-2/PGE2-mediated inflammatory reactions in intestinal epithelial cells.

Here, we hypothesize that Nrf2, activated by hyperoxia, participates in the regulation of ferroptosis and COX-2/PGE2-mediated inflammation in intestinal epithelial cells. To confirm this hypothesis, we systematically evaluate the effect of hyperoxia on oxidative damage and ferroptosis both in vitro and in vivo. We combine inhibitor intervention and protein expression analysis to determine the effect of Nrf2 on hyperoxia-induced ferroptosis and inflammation, as well as the underlying mechanism. The results of this study will provide a solid theoretical basis for preventing neonatal organ injury caused by hyperoxia treatment in clinical practice.

## Materials and methods

### Animals

The Animal Department of the Research and Development Center of Shengjing Hospital, China Medical University provided adult Sprague–Dawley (SD) rats, with a female to male mating ratio of 3 to 1, all neonatal SD rats were spontaneously delivered by females. All animal experiments were conducted in accordance with the national animal protection regulations of China and the guidelines of the Animal Protection and Use Committee of China Medical University (Approval No.: 2018PS178K). And all animal experiments should be carried out in accordance with the U.K. Animals (Scientific Procedures) Act, 1986 and associated guidelines, EU Directive 2010/63/EU for animal experiments or the National Research Council's Guide for the Care and Use of Laboratory Animals. Furthermore, reporting (not performance) of animal testing experiments should comply with the ARRIVE guidelines.

### Animal model and tissue harvest

Within 12 h after birth, newborn SD rats were randomly divided into two groups: a control group (FiO_2_ = 21%) and a hyperoxia group (FiO_2_ = 85%), each consisting of 8 rats. To avoid differences between the groups caused by oxygen poisoning, the female rats were exchanged every 24 h. On the 3rd, 7th, 10th, and 14th day after birth, rats were randomly selected from both groups and euthanized for intestinal tissue harvesting.

### Cell lines and cell cultivation

The NCM460 cells were cultured in RPMI 1640 incomplete medium (cat. no. Kgm31800-500 KeyGENBioTECH, Jiangsu, China), supplemented with 10% fetal bovine serum and 1% penicillin/streptomycin double antibiotic solution. The cells in the logarithmic growth phase were digested and passaged. In the control group, the cells were cultured in a ordinary incubator (FiO2 21%, 37 ℃, 5% CO2). In the hyperoxia group, after the cells were cultured in the ordinary incubator for 24 h, then were cultured in a hyperoxia incubator (FiO285%, 37 ℃, 5%CO2) for 24 h, 48 h, and 72 h, respectively. In the cells of the hyperoxia experimental group with antagonists and inhibitors, the culture medium added the drugs was changed every 24 h, and the cells were collected after the specified culture time was reached, and the specific concentrations were as follows, tBHQ (cat. no. S4990, Selleck; USA; 20 μM); ML385 (cat. no. S8790, Selleck; USA; 5 μM); Fer-1 (cat. no. A4371, APExBIO; USA; 1 μM); Celecoxib (cat. no. S1261, Selleck; USA; 50 μM); TG4-155 (cat. no. HY-18971, MCE; USA; 2 μM); CJ-42794 (cat. no. HY-10797, MCE; USA; 10 μM).

### Immunohistochemical (IHC) staining

#### Small intestinal tissue samples

A paraffin section of the intestinal tissue was taken, and dewaxing, antigen repair, blocking, and then were incubated with the primary antibodies: rabbit anti-recombinant divalent metal transporter 1(DMT1) (cat.no.20507-1-AP,Proteintech,Wuhan,China); rabbit anti-transferrin receptor(TFRC)(cat.no.A5865, Abclonal, Wuhan, China); rabbit anti-GPX4(cat.no.A13309,Abclonal,Wuhan,China); rabbit anti-FTH1(cat.no.A1144,Abclonal,Wuhan,China); rabbit anti-recombinant solute carrier family 7, member 11 (SLC7A11)(cat.no.A2413,Abclonal,Wuhan,China) were carried out at 4 ℃ overnight. Next the sections were in turn incubated with biotin-labeled goat anti-rabbit IgG and horseradish enzyme-labeled streptavidin working solution (cat. no. SP9001, Zhongshan Golden Bridge Biotech, Beijing, China) for 30 min. Images were taken using a light Microscope and a Nikon image acquisition system (Eclipse NI, Nikon, Tokyo, Japan). The expressions of proteins were analyzed using Image J 1.48 (National Institutes of Health) software.

#### Intestinal epithelial cells

NCM460cells were fixed on cover glass with 4% paraformaldehyde. Endogenous peroxidase of the cells was blocked with 3% H_2_O_2_ and 10% goat serum. Then as described above, the cells were in turn incubated with the primary antibodies (rabbit anti-DMT1, TFRC, GPX4, FTH1, SLC7A11, respectively), biotin-labeled goat anti-rabbit IgG and horseradish enzyme-labeled streptavidin working solution. Finally, DAB and hematoxylin were used for staining. Image acquisition and analysis were carried out as described above.

### Measurement of lipid peroxidation levels

The Lipid Peroxidation MDA Assay Kit (cat.no.BC0025,Solarbio,Beijing,China), the GSH Assay Kit (cat.no.BC1170,Solarbio,Beijing,China), and the Total SOD Assay Kit (cat.no.BC0175,Solarbio,Beijing,China) were used to analyse levels of malondialdehyde (MDA), GSH, and superoxide dismutase (SOD), respectively, following the kit instructions for all procedures.

### Detection of ROS level

NCM460 cells in the logarithmic growth phase were routinely digested and plated in 6-well plates. After the cells were attached, the cells in the control group were placed in an ordinary incubator and continued to be cultured for 24 h. The cells of the hyperoxia group were placed in the hyperoxia incubator and continued to be cultured for 24 h, 48 h and 72 h respectively, and then taken out, and the cells of the hyperoxia 72 h needed to be replaced once when the hyperoxia was 48 h. The cells were incubated with DCFH-DA probe in the dark at 37 ℃ and were observed by the fluorescence microscope, and the green fluorescence produced is the reactive oxygen species in the cell, and the brighter the green fluorescence, the higher the content of reactive oxygen species contained in the cell. The multimode microplate reader was adjusted to an excitation wavelength of 488 nm and a reception wavelength of 525 nm to measure the absorbance of each well in a 6-well plate. The higher the measured absorbance (OD) value, the more reactive oxygen species are produced.

### MMP assay

The cell culture is the same as the Detection of ROS level. The 5,5',6,6'-tetrachloro-1,1',3,3'-tetraethyl-benz imidazole carbon iodide (JC-1) fluorescent probe (cat. no. C2006, Beyotime, Shanghai, China) was used to detect MMP (mtΔΨ). The cells were incubate in a cell culture incubator at 37 ℃ for 20 min in the dark, wash off the probes and set aside on ice. Observation and photography were taken under a fluorescence microscope. The absorbance (OD) values of each well were measured with a multimode microplate reader. JC-1 monomer is green, JC-1 polymer is red, their maximum excitation wavelength is 490 nm, 525 nm, and the maximum emission wavelength is 530 nm and 590 nm, respectively.

### Cell mortality rate

Propidium iodide (PI) penetrates the membranes of dead cells, and can be inserted into double-stranded DNA, so the nuclei of dead cells are stained, but not living cells. And the cell mortality was measured by flow cytometry. Take out the treated cells, digest the cells with EDTA-free trypsin, pay attention to the supernatant of the original culture medium in the medium dish and store it in the centrifuge tube, terminate the digestion of the cells with the culture medium at the end of digestion, move the cell suspension to the centrifuge tube, resuspend the cells with pre-cooled PBS after centrifugation, repeat 2 times, transfer the cells to the flow cytometry tube, add PI solution, protect from light at room temperature for 15 min, insert the flow cytometer into ice, and go up to the flow cytometer for detection within 1 h.

### Western blot analysis

Protein was extracted from cells or intestinal tissue and quantified by BCA kit (cat.no.P0013C, Beyotime, Shanghai, China). The samples were transferred to 10% SDS-PAGE gel, then transferred to a polyvinylidene fluoride (PVDF) membranes and sealed using 5% skim milk. The membranes were incubated with primary antibodies: rabbit anti-DMT1; TFRC; GPX4; FTH1; SLC7A11; Nrf2 (cat.no. ab31163, Abcam, Cambridge, USA); COX-2 (cat.no.ab179800, Abcam, Cambridge, USA);TNF alpha (cat. no. 17590-1-AP, Proteintech, Wuhan, China); prostaglandin E receptor 4(EP4)(cat. no. 24895-1-AP, Proteintech, Wuhan, China); prostaglandin E receptor(EP2)(cat.no.ab167171, Abcam, Cambridge, USA) and mouse anti-IL-4 (cat.no.66142-1-Ig, Proteintech, Wuhan, China); IL-6 (cat.no.66146-1-Ig, Proteintech, Wuhan, China); β-actin (cat.no.66009-1-Ig, Proteintech, Wuhan, China) overnight at 4 ℃. Then the membranes were incubated with goat anti-rabbit or mouse IgG (cat.no.SA00001-2 or SA00001-1, Proteintech, Wuhan, China) and with an enhanced chemiluminescent substrate. Images were captured using Amersham Imager 680 (GE Healthcare Life Sciences, Pittsburgh, PA, USA). Band density values were calculated using ImageJ 6.0 (National Institutes of Health) and normalized to β-actin.

### Statistical analysis

Experimental data are presented as the mean ± SD. SPSS25.0 software (IBM Corp, Armonk, NY, USA) was used for statistical analysis, and GraphPad Prism 8.0 software (GraphPad Software, San Diego, CA, USA) was used to prepare charts. The unpaired *t*-test was used for comparison between groups. Two-factor analysis of variance was used for comparisons between multiple groups, followed by Bonferroni post-hoc tests.

## Results

### Hyperoxia induced oxidative stress and mitochondrial injury in vivo and in vitro

**Fig. 1:**
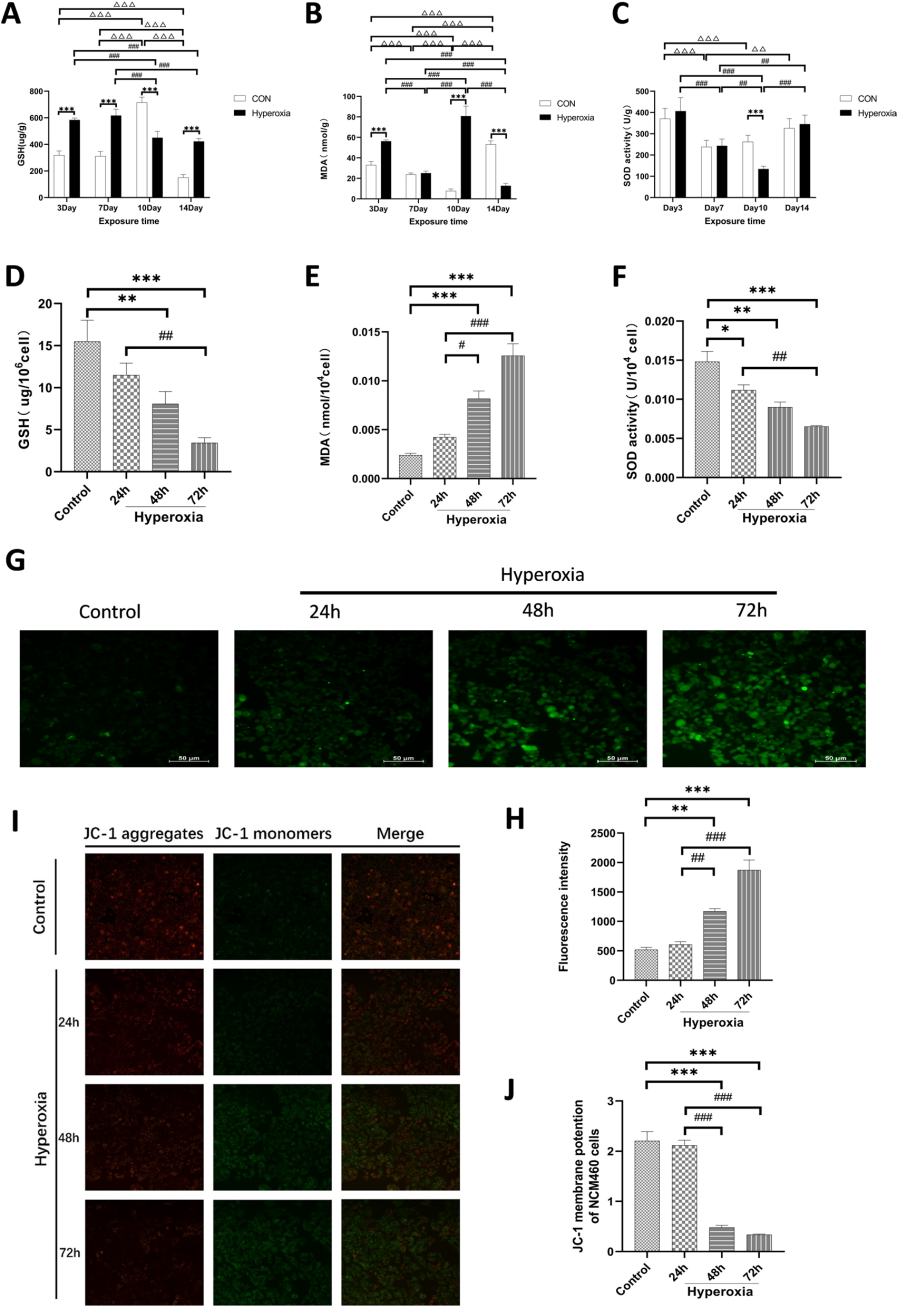
Effect of hyperoxia on oxidative stress and mitochondrial injury. The levels of GSH (**A**, **D**), MDA (**B**, **E**), and SOD (**C**, **F**) in vivo and in vitro were detected by corresponding kit. ROS level in intestinal epithelial cells was evaluated by DCFH-DA (**G**). Quantification of ROS images (**H**). MMP level in intestinal epithelial cells was evaluated by JC-1 (**I**). Quantification of MMP images (**J**). (The hyperoxia group was compared with the control group:*P < 0.05, **P < 0.01, ***P < 0.001; Expression in the control group was compared between different time points, ^△^*P* < 0.05, ^△△^*P* < 0.01, ^△△△^*P* < 0.001; Expression in the hyperoxia group was compared between different time points, ^#^*P* < 0.05, ^##^*P* < 0.01, ^###^*P* < 0.001,* n* > 8)

High concentrations of oxygen can disrupt the balance of ROS. Therefore, we first investigated hyperoxia-induced oxidative stress in the intestinal tissue of newborn rats and intestinal epithelial cells. Compared with the control group, after hyperoxia treatment, the GSH content and the activity of SOD decreased significantly, MDA content increased in rats, these differences were most significant on day 10 in vivo (*P* < 0.001; Fig. 1A–C). We also evaluated hyperoxia-induced oxidative stress in vitro. The results showed that hyperoxia led to a decrease in GSH and SOD, and an increase in MDA, and after 72 h of hyperoxia treatment, the change in the oxidative stress index was the most significant (*P* < 0.01 or *P* < 0.001; Fig. 1D–F). Subsequently, we evaluated the ROS level in intestinal epithelial cells. Compared to the control group, the ROS level increased at 48 h and peaked at 72 h (*P* < 0.01 or *P* < 0.001; Fig. 1G, H). Finally, we explored hyperoxia-induced mitochondrial injury using the JC-1 kit. The results showed that the MMP level in intestinal epithelial cells decreased significantly at 48 h and 72 h (*P* < 0.001; Fig. 1I, J). In vitro and in vivo experiments indicated that hyperoxia increased the release of ROS, resulting in oxidative stress and mitochondrial injury in a time-dependent manner.

### Hyperoxia induced ferroptosis in vivo and in vitro

**Fig. 2:**
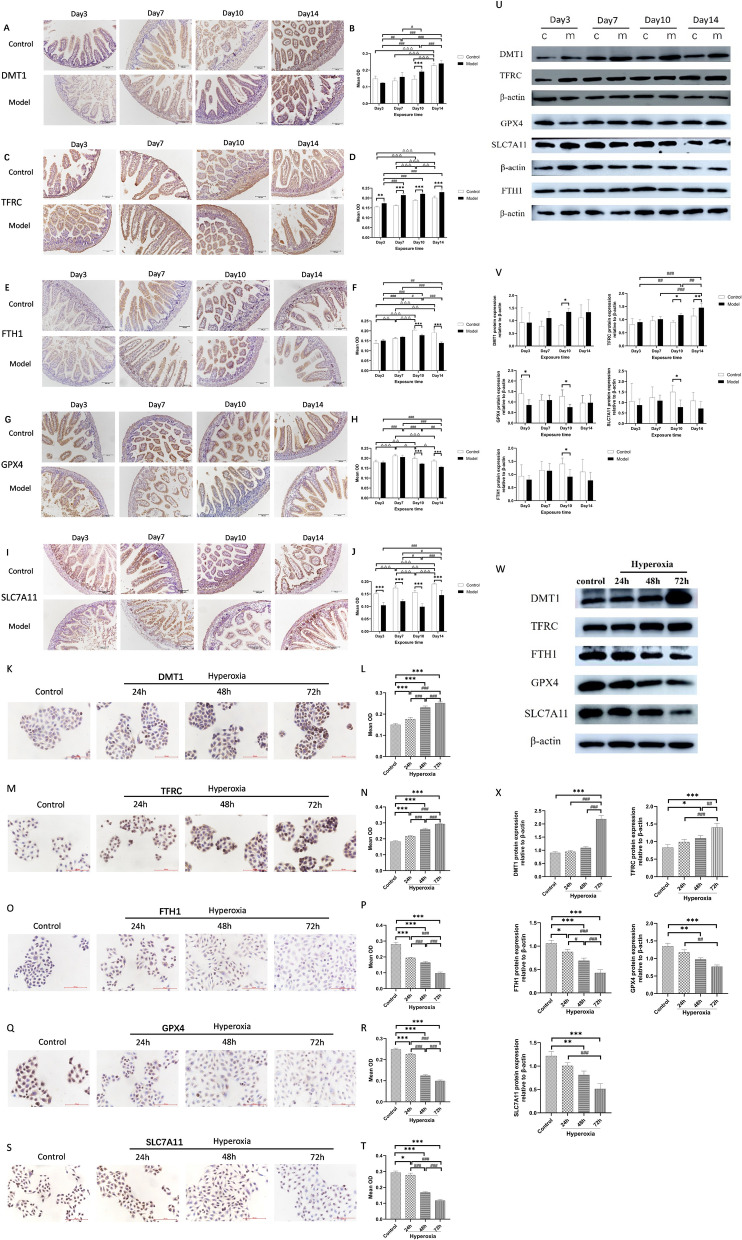
Effect of hyperoxia on ferroptosis-related proteins in vivo and vitro. In vivo: The expressions of DMT1 (**A**), TFRC (**C**), FTH1 (**E**), GPX4 (**G**), and SLC7A11 (**I**) were evaluated by IHC. Quantification of IHC images of DMT1 (**B**), TFRC (**D**), FTH1 (**F**), GPX4 (**H**) and SLC7A11 (**J**). In vitro: The expressions of DMT1 (**K**), TFRC (**M**), FTH1 (**O**), GPX4 (**Q**), and SLC7A11 (**S**) were evaluated by IHC. Quantification of IHC images of DMT1 (**L**), TFRC (**N**), FTH1 (**P**), GPX4 (**R**), and SLC7A11 (**T**). In vivo: The expressions of ferroptosis-related proteins were evaluated by Western blot (**U**). Quantification of bands of DMT1, TFRC, GPX4, SLC7A11 and FTH1 (**V**). In vitro: The expressions of ferroptosis-related proteins were evaluated by western blot (**W**). Quantification of bands of DMT1, TFRC, FTH1, GPX4 and SLC7A11 (**X**). (The hyperoxia group was compared with the control group:*P < 0.05, **P < 0.01, ***P < 0.001; Expression in the control group was compared between different time points, ^△^*P* < 0.05, ^△△^*P* < 0.01, ^△△△^*P* < 0.001; Expression in the hyperoxia group was compared between different time points, ^#^*P* < 0.05, ^##^*P* < 0.01, ^###^*P* < 0.001,* n* > 8)

To investigate the impact of hyperoxia on ferroptosis, we examined the expression of ferroptosis-related proteins using IHC and western blot in both in vivo and in vitro settings. IHC results from rat intestinal tissue demonstrated that compared to the control group, the expressions of DMT1 and TFRC increased (*P* < 0.01 or *P* < 0.001; Fig. 2A–D), while GPX4 decreased from day 7 to day 14 (*P* < 0.01 or *P* < 0.001; Fig. 2G, H), but FTH1 increased on day 7 and decreased on day 10 and day 14(*P* < 0.01 or *P* < 0.001; Fig. 2E, F), while SLC7A11 decreased only on day 14 (*P* < 0.001; Fig. 2I, J). However, western blot results showed compared to the control group, the expression of DMT1 increased, while GPX4, SLC7A11, and FTH1 decreased on day 10, and TFRC significantly increased on day 10 and day 14 (*P* < 0.05, *P* < 0.01 or *P* < 0.001; Fig. 2U, V). We also conducted protein expression analyses in vitro. The results of IHC indicated a significant increase in the expressions of DMT1 and TFRC from 24 to 72 h, whereas FTH1, GPX4 and SLC7A11 showed significant decreases compared to the control group (*P* < 0.05 or *P* < 0.001; Fig. 2K–T). Consistent with the IHC findings, western blot results showed that DMT1 and TFRC significantly increased at 72 h, whereas FTH1, GPX4 and SLC7A11 showed a decrease in expression with increasing hyperoxia treatment time (*P* < 0.05, *P* < 0.01, or *P* < 0.001; Fig. 2W, X). Taken together, these findings suggest that hyperoxia-induced ferroptosis occurred in the intestinal tissue of neonatal rats and epithelial cells.

### Nrf2 mediated the regulation of hyperoxia-induced ferroptosis

**Fig. 3:**
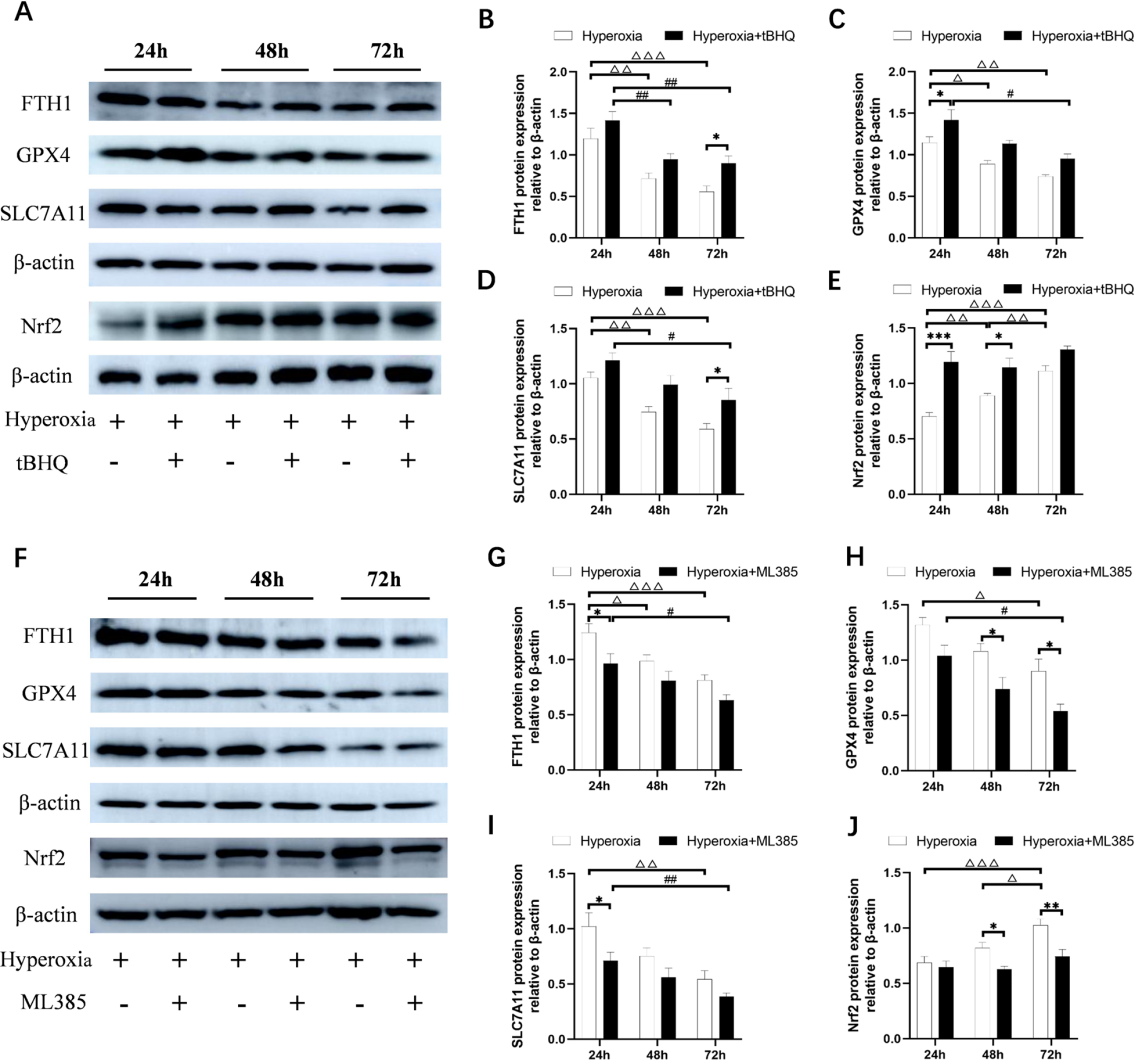
Effect of Nrf2 on hyperoxia-induced ferroptosis in vitro. After up-regulating Nrf2, the expressions of ferroptosis-related proteins were evaluated by western blot (**A**). Quantification of bands of FTH1 (**B**), GPX4 (**C**), SLC7A11 (**D**), and Nrf2 (**E**). After down-regulating Nrf2, the expressions of ferroptosis-related proteins were evaluated by western blot (**F**). Quantification of bands of FTH1 (**G**), GPX4 (**H**), SLC7A11 (**I**), and Nrf2 (**J**). (The treatment group was compared with the hyperoxia group:*P < 0.05, **P < 0.01, ***P < 0.001; Expression in the hyperoxia group was compared between different time points, ^△^*P* < 0.05, ^△△^*P* < 0.01, ^△△△^*P* < 0.001; Expression in the treatment group was compared between different time points, ^#^*P* < 0.05, ^##^*P* < 0.01, ^###^*P* < 0.001,* n* > 8)

To investigate the effect of Nrf2 on hyperoxia-induced ferroptosis, we used the Nrf2 agonist tBHQ and inhibitor ML385 to up-regulate and down-regulate Nrf2, respectively. As hyperoxia time was prolonged, the levels of FTH1, GPX4, and SLC7A11 gradually decreased, while the expression of Nrf2 gradually increased (*P* < 0.05, *P* < 0.01 or *P* < 0.001; Fig. 3A–J). Compared to the hyperoxia group, the expression of FTH1, GPX4, SLC7A11 and Nrf2 significantly increased (*P* < 0.05 or *P* < 0.01; Fig. 3A–E) after treatment with tBHQ, but as expected, the levels of FTH1, GPX4, and SLC7A11 further decreased and the expression of Nrf2 was inhibited after treatment with ML385 (*P* < 0.05 or* P* < 0.01; Fig. 6F–J). These findings suggest that Nrf2 plays a role in mediating the regulation of hyperoxia-induced ferroptosis.

### Hyperoxia activated COX-2/PGE2/EP signaling pathway and induced inflammation

**Fig. 4:**
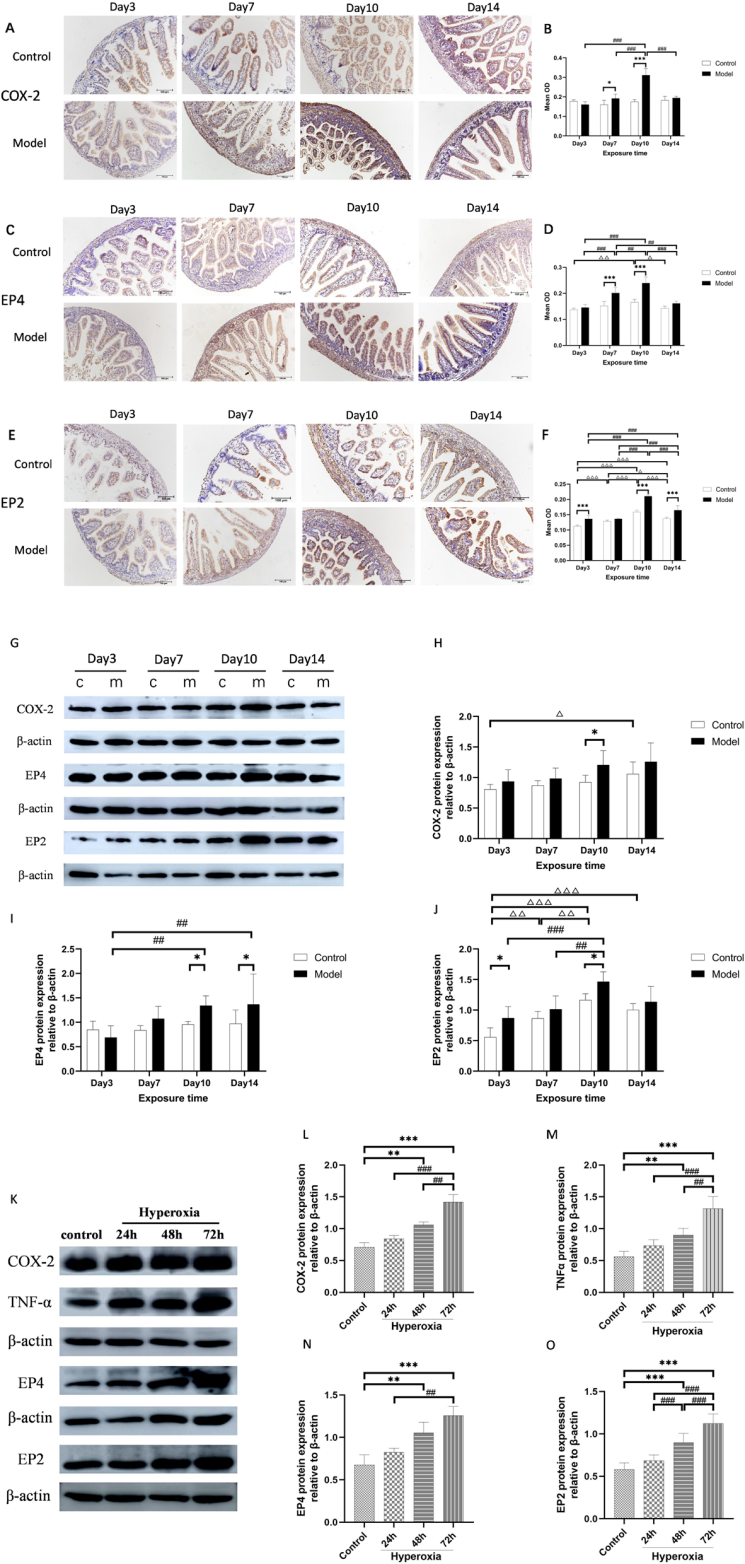
Effect of hyperoxia on COX-2/PGE2/EP in vivo and in vitro. In vivo: The expressions of COX-2 (**A**), EP2 (**C**), and EP4 (**E**) proteins were evaluated by IHC. Quantification of COX-2 (**B**), EP2 (**D**), and EP4 (**F**). The expressions of COX-2, EP4, and EP2 were evaluated by western blot (**G**). Quantification of bands of COX-2 (**H**), EP4 (**I**), and EP2 (**J**). In vitro: The expressions of COX-2, TNF-α, EP4 and EP2 proteins were evaluated by western blot (**K**). Quantification of bands of COX-2 (**L**), TNF-α (**M**), EP4 (**N**), and EP2 (**O**). (The hyperoxia group was compared with the control group:*P < 0.05, **P < 0.01, ***P < 0.001; Expression in the control group was compared between different time points, ^△^*P* < 0.05, ^△△^*P* < 0.01, ^△△△^*P* < 0.001; Expression in the hyperoxia group was compared between different time points, ^#^*P* < 0.05, ^##^*P* < 0.01, ^###^*P* < 0.001,* n* > 8)

To investigate the effect of hyperoxia on the COX-2/PGE2/EP signaling pathway, we first assessed the expression of COX-2, EP2, and EP4 in the intestinal tissue of neonatal rats. The IHC results showed that compared to control group, in the hyperoxia group the expression of COX-2 increased on day 7, and EP4 increased on day 7 and day 10, and the expression of EP2 increased on day 3, day 10 and day 14, with the highest expression levels observed on day 10 (*P* < 0.05 or* P* < 0.001; Fig. 4A–F). The western blot results confirmed these findings, with the expression of COX-2, EP4, and EP2 increasing to varying degrees under hyperoxia (*P* < 0.05, *P* < 0.01, or* P* < 0.001; Fig. 4G–J), indicating the activation of the COX-2/PGE2/EP2 signaling pathway in hyperoxia. To further confirm this pathway, we used intestinal epithelial cells and observed that with an increase in hyperoxia exposure time, the expression of COX-2, EP4, and EP2 increased gradually (*P* < 0.01 or* P* < 0.001; Fig. 4K, L, N–O). Moreover, the level of TNF-α significantly increased at 48 h and 72 h (*P* < 0.01; Fig. 4K and M), suggesting that hyperoxia activates the COX-2/PGE2/EP signaling pathway and induces inflammation.

### Hyperoxia induced inflammation via COX-2/PGE2/EP signaling pathway

**Fig. 5:**
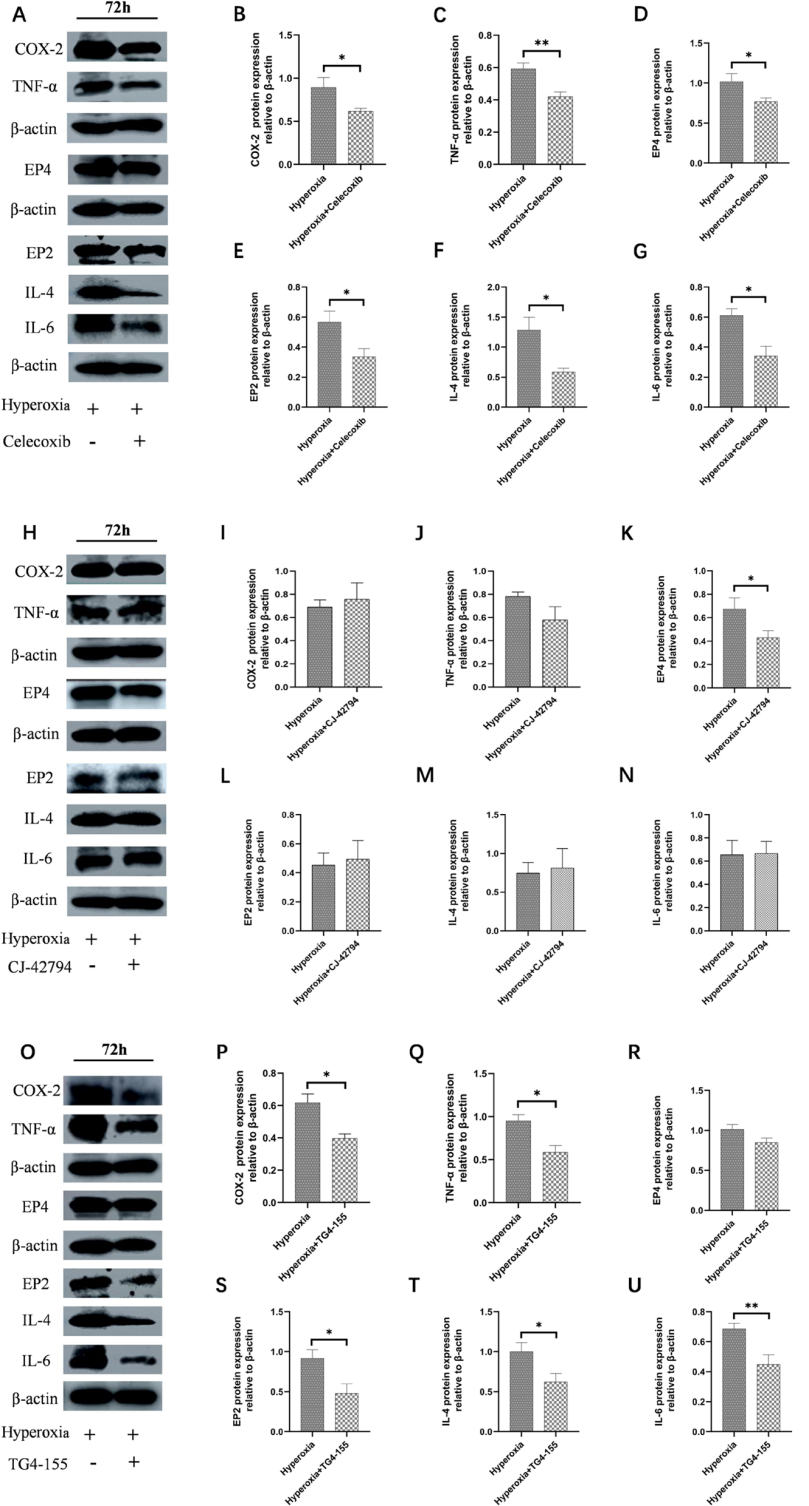
Effect of EP4/EP2 on COX-2/PGE2/EP and inflammation under hyperoxia. The expressions of COX-2/PGE2/EP and inflammation-related proteins were evaluated by western blot (**A**). Quantification of bands of COX-2 (**B**), TNF-α (**C**), EP4 (**D**), EP2 (**E**), IL-4 (**F**), and IL-6 (**G**). After down-regulating EP4, the expressions of COX-2/PGE2/EP and inflammation-related proteins were evaluated by western blot (**H**). Quantification of bands of COX-2 (**I**), TNF-α (**J**), EP4 (**K**), EP2 (**L**), IL-4 (**M**), and IL-6 (**N**). After down-regulating EP2, the expressions of related proteins were evaluated by western blot (**O**). Quantification of bands of COX-2 (**P**), TNF-α (**Q**), EP4 (**R**), EP2 (**S**), IL-4 (**T**) and IL-6 (**U**). (The treatment group was compared with the hyperoxia group:^*^P < 0.05, ^**^P < 0.01, ***P < 0.001,* n* > 8)

To investigate the impact of COX-2 on inflammation in intestinal epithelial cells under hyperoxia, the cells were exposed to hyperoxia for 72 h and then treated with the COX-2 inhibitor Celecoxib. The results showed that the expression levels of COX-2, EP4, and EP2 were reduced in the Celecoxib-treated group compared to the hyperoxia group (*P* < 0.05; Fig. 5A, B, D, E), indicating that the COX-2 pathway was inhibited. Furthermore, the levels of the inflammatory factors TNF-α, IL-4, and IL-6 were significantly reduced (*P* < 0.05 or* P* < 0.01; Fig. 5A, C, F, G). These findings suggest that hyperoxia induces inflammation via the COX-2/PGE2/EP signaling pathway.

### COX-2 plays a pro-inflammatory role during hyperoxia via the EP2 receptor

To determine the COX-2 receptor involved in hyperoxia-induced inflammation, we treated the cells with EP4 inhibitor CJ-42794 and EP2 inhibitor TG4-155 to down-regulate EP4 and EP2, respectively. The results demonstrated that compared to the hyperoxia group EP4 markedly decreased (*P* < 0.01; Fig. 5K), but there was no statistical difference in the expression of COX, EP2, TNF-α, IL-4 and IL-6 after adding CJ-42794 (*P* > 0.05; Fig. 5H–J, L–N). However, after inhibiting EP2, the expression of COX, EP2, TNF-α, IL-4, and IL-6 markedly decreased (*P* < 0.05 or* P* < 0.01; Fig. 5O–U), except for EP4 (*P* > 0.05; Fig. 5R). These results suggest that during hyperoxia, COX-2 plays a pro-inflammatory role through the EP2 receptor rather than the EP4 receptor.

### Hyperoxia-induced oxidative damage regulated inflammation through ferroptosis

**Fig. 6:**
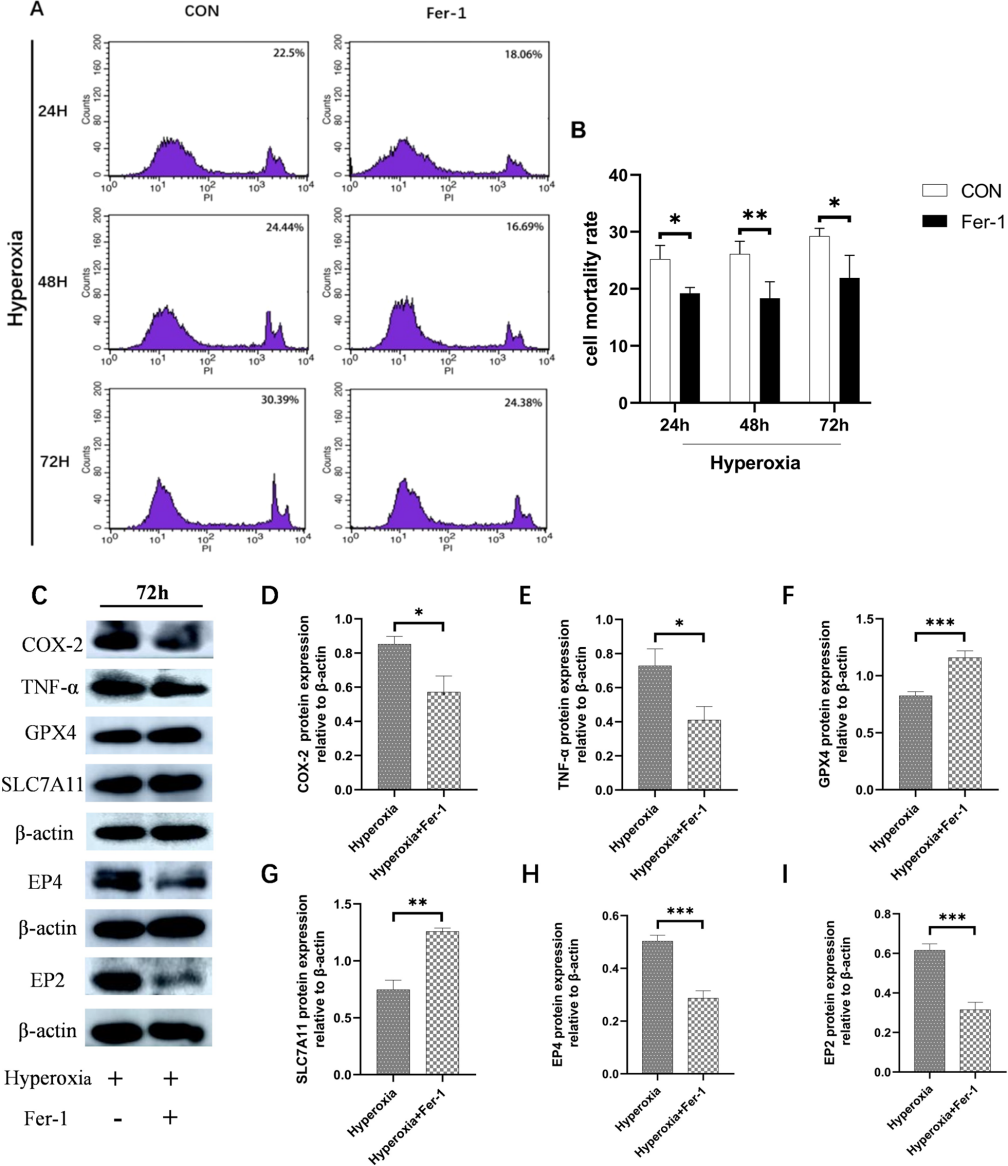
Effect of ferroptosis on COX-2/PGE2/EP in hyperoxia. Cell mortality rate was evaluated by flow cytometry (**A**). Quantification of cell mortality rate (**B**). The expressions of COX-2/PGE2/EP and ferroptosis-related proteins were evaluated by western blot (**C**). Quantification of bands of COX-2 (**D**), TNF-α (**E**), GPX4 (**F**), SLC7A11 (**G**), EP4 (**H**), and EP2 (**I**). (The treatment group was compared with the hyperoxia group:^*^P < 0.05, ^**^P < 0.01, ***P < 0.001,* n* > 8)

To elucidate the role of ferroptosis in hyperoxia-induced inflammation, we treated intestinal epithelial cells with the ferroptosis inhibitor Fer-1. Flow cytometry results showed that with increasing exposure time to hyperoxia, the cell death rate progressively increased, and after inhibiting ferroptosis, the cell death rate decreased significantly (*P* < 0.05 or *P* < 0.01; Fig. 6A, B). As cell damage was most pronounced at 72 h, we chose this time point for further experiments. We observed that adding Fer-1 to hyperoxia cells increased the expression of GPX4 and SLC7A11, but decreased the expression of COX-2, TNF-α, EP4, and EP2 (*P* < 0.05, *P* < 0.01or* P* < 0.001; Fig. 6C-I), indicating that Fer-1 relieved hyperoxia-induced oxidative damage and suppressed hyperoxia-induced inflammation. These findings suggest that hyperoxia-induced oxidative damage regulates inflammation through ferroptosis.

## Discussion

The hyperoxia toxicity can damage the intestinal barrier of newborn rats, resulting in impaired intestinal development and function (Chou and Chen 2017). As a result, long-term oxygen therapy can cause nutritional absorption disorders and even restrict the growth and development of children. In this study, we have confirmed that ferroptosis plays a significant role in hyperoxia-induced injury of the intestinal tissue, both in vitro and in vivo. Additionally, we have discovered that hyperoxia activates Nrf2 to regulate ferroptosis and mediate inflammatory reactions via the COX-2/PGE2/EP2 pathway. These findings can serve as a new experimental basis for preventing neonatal organ injury resulting from hyperoxia treatment in clinical practice.

In this study, we developed a hyperoxia rat model by exposing newborn rats to hyperoxia on day 3, day 7, day 10, and day 14 after birth to investigate the mechanism of intestinal injury caused by hyperoxia. Excessive ROS can stimulate pathological redox signals leading to oxidative stress (Zhu et al. 2022). Cells develop their own antioxidant mechanisms, including several antioxidant enzymes such as the SOD enzyme family, glutathione peroxidase (GPX), and non-enzyme substances such as GSH to combat oxidative stress (Jelic et al. 2021). Our results showed that compared to the control, SOD and GSH levels increased on day 3 and day 7, decreased significantly on day 10, and then increased again on the 14th day. Conversely, MDA, the end-product of lipid peroxidation, showed an opposite trend (Leon and Borges 2020). Therefore, we hypothesized that the early increase of GSH and SOD might be a response to oxidative stress induced by hyperoxia. As the exposure time to hyperoxia increased, the antioxidant capacity of the intestinal tissue of neonatal rats decreased, as evidenced by the reduction in GSH and SOD levels and the increase in MDA levels on the 10th day of hyperoxia. Additionally, hyperoxia led to the accumulation of ROS, causing oxidative stress. Mitochondria were identified as the primary source of intracellular ROS, and excessive ROS can lead to mitochondrial damage. Our findings indicate that hyperoxia causes a decrease in mitochondrial membrane potential, leading to oxidative stress and reduction of the cellular antioxidant capacity. This results in the accumulation of ROS in cells, leading to mitochondrial and cellular damage. These characteristics are also observed in ferroptosis, which leads us to speculate that ferroptosis may contribute to the intestinal oxidative damage induced by hyperoxia.

Ferroptosis is an iron and oxidation-dependent form of cell death, and iron is essential for the accumulation of lipid peroxide and the onset of ferroptosis (Zhang et al. 2022b). The balance of iron metabolism is maintained through the input, output, and storage of iron ions (Fan et al. 2022). Iron input proteins include transferrin, TFRC, and DMT1, while iron output proteins include ferroportin 1 (Fpn1) (Dutt et al. 2022). Currently, it is believed that the occurrence of ferroptosis is related to abnormalities in iron metabolism, such as GPX4 inactivation, cystine/glutamate reverse transport system (System Xc-) inhibition, and lipid peroxidation, ultimately leading to an imbalance in ROS homeostasis and cell death (Su et al. 2019; Ursini and Maiorino 2020). In both in vivo and in vitro experiments, we found that hyperoxia increased DMT1 and TFRC, and decreased Nrf2 targeting gene FTH1 as well as the antioxidant protein GPX4 and SLC7A11, indicating the occurrence of ferroptosis. Nrf2 plays a crucial role in maintaining normal redox homeostasis and in mediating other metabolic pathways, including protease balance, iron/heme metabolism, lipid metabolism, and cell apoptosis (He et al. 2020a, 2020b). So Nrf2 was involved in regulating the iron transporter and iron storage proteins (Wang et al. 2022). And Nrf2 could directly or indirectly regulate the expression and function of GPX4 (Dodson et al. 2019). In this study, we used the Nrf2 agonist tBHQ and its inhibitor ML385 to up/down-regulate its expression, respectively. The results showed that up-regulation of Nrf2 in intestinal epithelial cells inhibited the occurrence of ferroptosis under hyperoxia and played a protective role during cell injury, while down-regulation of Nrf2 had the opposite effect. These results suggest that a lot of Nrf2 protected cells from damage by inhibiting ferroptosis in hyperoxia.

In ferroptosis, inflammatory mediators are produced by lipid peroxidation and AA metabolism, such as COX-2, which is also the key rate-limiting enzyme in the synthesis of PGs (Dissanayake et al. 2022; Zhao et al. 2022). In this study, we detected COX-2 and its downstream molecules-EP2 and EP4, namely the two receptor subtypes of PGE2. We found that hyperoxia activated the COX-2/PGE2 pathway and up-regulated the expression of the pro-inflammatory factor TNF-α, suggesting that hyperoxia leads to ferroptosis in parallel with inflammatory damage in vivo and in vitro. In ferroptosis COX-2 is a key marker and increased significantly (Lin et al. 2022). To confirm the role of the COX-2/PGE2/EP pathway in hyperoxia-induced intestinal epithelial cell inflammation, we first added the COX-2 inhibitor Celecoxib to the intestinal epithelial cells. As expected, COX-2 inhibition reduced the expression of downstream receptors EP2 and EP4, and partially inhibited the inflammation caused by hyperoxia in intestinal epithelial cells. Previous studies have shown that PGE2 causes acute inflammation by relaxing vascular smooth muscle cells through the EP2/EP4 signal pathway (Yao et al. 2009). PGE2 also promotes Th1 cell differentiation, Th17 cell proliferation, and IL-22 production of Th22 cell in vitro through EP2 and EP4 receptors (Lee et al. 2019). This shows that EP2 and EP4 receptors play a significant role in inflammation. To explore the specific downstream receptors, EP2 and EP4 were inhibited, respectively. Interestingly, while the addition of EP2 inhibitor TG4-155 successfully inhibited the expression of COX-2 and EP2 in intestinal epithelial cells and reduced the levels of inflammation-related factors TNF-α, IL-4 and IL-6, the addition of EP4 inhibitor had no significant effect. These results indicated that the continuous up-regulation of COX-2 during hyperoxia increased the level of PGE2, which promoted inflammation through the EP2 receptor rather than the EP4 receptor.

Many studies have reported that the inflammation pathways are involved in ferroptosis, and ferroptosis can regulate the activity of these pathways, affecting cellular biological function (Chen et al. 2023). A recent study showed that ferroptosis and inflammation are closely linked and together realize physiological and pathological functions. That anti-inflammatory treatment inhibited ferroptosis, and ferroptosis inhibitor Fer-1 inhibited COX-2, in other words, ferroptosis and inflammation may also complement each other (Zhang et al. 2023). In this study Fer-1relieved hyperoxia-induced oxidative damage and also reduced the expression of COX-2, EP4, EP2 and TNF-α, and reduced inflammation in intestinal epithelial cells in hyperoxia. Our findings were similar to a previous study in which Fer-1 decreased the level of ROS and alleviated inflammation (Zhang et al. 2018). So we conclude that in hyperoxia ferroptosis deteriorated oxidative damage and inflammation. Inhibition of ferroptosis may be a new direction for the treatment of hyperoxia intestinal inflammatory injury in the future.

## Conclusions

In conclusion, our study has revealed for the first time that ferroptosis is involved in the hyperoxia-induced intestinal oxidative damage both in vivo and in vitro. Furthermore, hyperoxia-activated Nrf2 regulates ferroptosis in intestinal epithelial cells and intervenes in inflammation through the COX-2/PGE2/EP2 pathway. These findings provide important experimental basis and theoretical basis for future clinical prevention and therapeutic approaches for neonatal intestinal injury caused by hyperoxia. Next, we will further study how COX-2/EP2 affects inflammation induced by hyperoxia.

## Data Availability

The authors confirm that the data supporting the findings of this study are available within the article [and/or its supplementary material.
